# Systematic Conservation Planning in the Face of Climate Change: Bet-Hedging on the Columbia Plateau

**DOI:** 10.1371/journal.pone.0028788

**Published:** 2011-12-08

**Authors:** Carrie A. Schloss, Joshua J. Lawler, Eric R. Larson, Hilary L. Papendick, Michael J. Case, Daniel M. Evans, Jack H. DeLap, Jesse G. R. Langdon, Sonia A. Hall, Brad H. McRae

**Affiliations:** 1 School of Forest Resources, University of Washington, Seattle, Washington, United States of America; 2 School of Aquatic and Fishery Sciences, University of Washington, Seattle, Washington, United States of America; 3 Department of Biology, University of Washington, Seattle, Washington, United States of America; 4 The Nature Conservancy, Wenatchee, Washington, United States of America; 5 The Nature Conservancy, Seattle, Washington, United States of America; University of Western Ontario, Canada

## Abstract

Systematic conservation planning efforts typically focus on protecting current patterns of biodiversity. Climate change is poised to shift species distributions, reshuffle communities, and alter ecosystem functioning. In such a dynamic environment, lands selected to protect today's biodiversity may fail to do so in the future. One proposed approach to designing reserve networks that are robust to climate change involves protecting the diversity of abiotic conditions that in part determine species distributions and ecological processes. A set of abiotically diverse areas will likely support a diversity of ecological systems both today and into the future, although those two sets of systems might be dramatically different. Here, we demonstrate a conservation planning approach based on representing unique combinations of abiotic factors. We prioritize sites that represent the diversity of soils, topographies, and current climates of the Columbia Plateau. We then compare these sites to sites prioritized to protect current biodiversity. This comparison highlights places that are important for protecting both today's biodiversity and the diversity of abiotic factors that will likely determine biodiversity patterns in the future. It also highlights places where a reserve network designed solely to protect today's biodiversity would fail to capture the diversity of abiotic conditions and where such a network could be augmented to be more robust to climate-change impacts.

## Introduction

Biological reserves were originally established on an opportunistic, ad hoc basis. As a result, many of the early reserves were situated in remote, high-elevation regions on less productive soils [1]. However, in the past 30 years, conservation scientists have attempted to correct this bias through systematic planning methods that better represent species, communities, and ecosystems [2]. These methods involve selecting land for reserves to maximize representation of conservation elements (e.g., priority species, ecosystems, or other aspects of biodiversity) [3] while minimizing the number of sites required or the costs to conserve them [2], [4].

Although this general approach to conservation planning can efficiently represent particular aspects of current biodiversity in reserve networks, it may fail to protect biodiversity in a changing climate [5]. There is already ample evidence that species are responding to recent changes in climate with significant shifts in their distributions [5], [6], [7], [8], and more extensive shifts are projected for the next century [9], [10]. As a result, species may lose protection as their ranges shift out of current reserve boundaries [11], [12], [13], [14], [15]. Planning approaches that focus on current species occurrences may therefore fail to protect biodiversity in the future.

Many researchers have suggested increasing the area of protected land as a strategy for conserving biodiversity in a changing climate [16], [17], [18]. Some strategies for placement of new reserves include protecting corridors between reserves to facilitate species range shifts [16], [18], protecting refugia (i.e., areas that are projected to change the least under multiple climate-change scenarios) [19], [20], [21], or planning reserves based on projections of future species distributions [13]. However, the uncertainty inherent in forecasting future climatic changes and the associated responses of biodiversity reduces the level of confidence one can have in the effectiveness of a reserve network based on model projections [22].

To avoid these uncertainties, researchers have suggested protecting the abiotic variability of a landscape as an alternative reserve-selection method [23], [24]. The abiotic variables in this approach often include slope, elevation, soil productivity, and climate. Facets, or unique combinations of abiotic factors, have been shown to represent regions of unique biota [23], [25], [26]. Therefore, a set of reserves that includes the breadth of abiotic variability may encompass the scope of biotic variability as well [27]. However, in contrast to biotic elements, land-facet (non-climatic abiotic facet) elements will be largely unaltered by climate change [28]; moreover, protecting the maximum range of current climate variability may protect the variability of future climates as well [29]. Therefore, conserving the range of land attributes and climate variability preserves a diversity of conditions that will likely support a diversity of species and ecosystems presently and under the future conditions that we might not be able to anticipate.

Like most climate-adaptation strategies, the idea of protecting abiotic facets is not new but rather a new application of an existing concept. Several studies have used abiotic elements in the absence of biodiversity data as surrogates for coarse-filter conservation elements in conservation planning [26], [30], [31]. However, these studies did not explicitly demonstrate the use of systematically classified abiotic facets to plan for biodiversity in a changing climate.

Using the Columbia Plateau ecoregion in the northwestern United States as a case study, we demonstrate how abiotic facets can be integrated into the conservation-planning process as a method for addressing climate change in traditional planning. Our primary objectives were to (1) compare abiotic-facet-based reserves to reserves selected to protect traditional elements; (2) identify tradeoffs between the two approaches and highlight regions that could make a biodiversity-based network more robust to the uncertain impacts of climate change; and (3) explore the additional utility of abiotic-facet-based planning to locate priority regions for restoration. The Columbia Plateau ecoregion is an appropriate case study for testing this approach because the area is home to a number of threatened and endangered species, consists primarily of privately-owned agricultural land, and has an arid climate that is sensitive to climatic changes.

## Materials and Methods

### Study area

**Figure 1:**
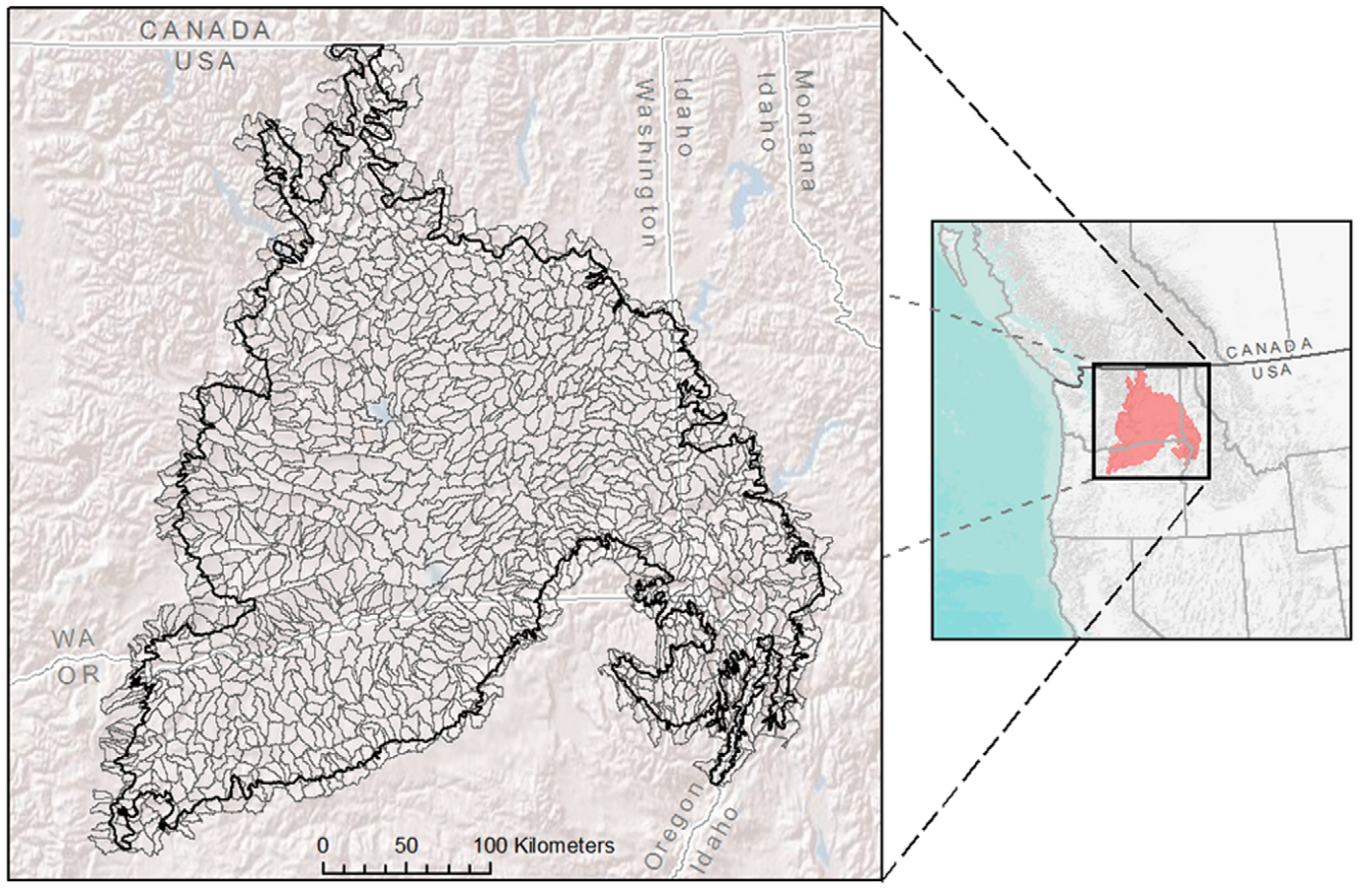
Columbia Plateau ecoregion. The map depicts 1200 hydrologic unit codes (HUC-12) that were used as conservation planning units in the present study.

The Columbia Plateau ecoregion in the northwestern United States is an 11.2 million hectare area, bounded by the Rocky Mountains to the east, the Cascade Mountains to the west, and the Blue Mountains to the southeast (Figure 1). This unique landscape includes broad plateaus bordered by steep columnar basalt coulees, gentle slopes, scattered pothole lakes and vernal pools, and the Columbia and Snake River systems. The soil is mostly deep loam, with some sandy, shallow, stony, or alkali areas. Because it sits in the rain shadow of the Cascade Mountains, the plateau is relatively dry. It is dominated by sagebrush steppe and is home to over 200 vulnerable plant and animal species, including the sharp-tailed grouse (*Tympanuchus phasianellus*) and pygmy rabbit (*Brachylagus idahoensis*).

In addition to being an important area for biological diversity, the Columbia Plateau is also a major agricultural region. The landscape has been heavily altered by human use through farming, grazing, altered fire regimes, housing development, and hydropower [32]. The majority of the remaining native vegetation is now found in steep canyons and coulees and on shallow scabland soils. The combination of biological richness and anthropogenic threats make this region a high priority area for conservation. Consequently, the region has been the focus of several previous pilot planning studies [33], [34], [35].

### Defining abiotic facets

We described the abiotic diversity of the Columbia Plateau ecoregion using two topographic variables, three soil variables, and four climatic variables. We used these variables in cluster analysis to identify abiotic facets. Below, we describe the variables that defined the facets, the clustering approach, and a simple test to investigate the potential ecological relevance of the facets.

Data for all abiotic variables were aggregated to a 240-m grid. We derived elevation and slope from United States Geological Survey Digital Elevation Model (DEM) datasets for the states of Idaho, Washington, and Oregon (Table S1). We chose three soil characteristics that together indicate potential vegetative productivity: soil depth, available water storage, and texture or particle size. We used soil data from the finer-resolution Soil Survey Geographic Database (SSURGO) for regions with information available for these soil variables and data from the State Soil Geographic Database (STATSGO) for regions without finer-resolution information (Table S1). SSURGO soil data are available at resolutions between 1∶12,000 and 1∶63,360 and are created from digitized soil survey maps in combination with interpolation from measured soil samples and other landscape features that can be used to estimate soil characteristics. STATSGO data are available at 1∶240,000 resolutions and are compiled into seamless polygons through estimates based on other topographic, climatic, and geomorphic features. The measure of soil depth that we used represents the distance from the top of the soil to the base of the soil horizon. As a measure of texture or particle size, we used the percentage of soil, by weight, that is able to pass through a number 40 sieve with a 0.42 mm square opening. For available water storage (AWS), we used the weighted average of the volume of water that the soil can store and that is available to plants, between 0 and 150 centimeters depth. In SSURGO datasets, AWS is determined either by direct measurements, if available, or estimates based on other known properties of the soil, often including soil texture. The resolution of the soils data, any inherent error in those data due to interpolation or sampling, as well the difference in resolution resulting from the combination of the SSURGO and STATSGO data sources likely influenced the location and size of contiguous land facets in some regions. Although geologic variables have been used in the characterization of land facets in previous studies, they are generally used as surrogates for unavailable soil productivity data [24], [36], [37]. Thus, we did not include geologic variables.

To represent climate variability in the study area, we chose four climate variables: mean maximum temperature during the warmest month (July), mean minimum temperature during the coldest month (January), mean total precipitation for the wettest month (December), and mean total precipitation for the driest month (July). Climate data were taken from modeled 1/16^th^ degree resolution climate surfaces generated for the Columbia Basin and represented an averaged time period from 1915–2006 (Table S1). Although we could have used more biologically meaningful climate variables, many climate variables are highly correlated and it is likely that these simpler variables captured the major climate patterns.

We identified abiotic facets in the Columbia Plateau ecoregion by clustering the preceding normalized variables into groups that minimized variation for the nine topographic, soil, and climate variables. We also identified clusters of climate facets (climate variables only) and land facets (topographic and soil variables only) to explore the effects of variable choice on our prioritization results (Text S1, Figure S1). We used the K-means clustering algorithm, a non-hierarchical clustering approach, to classify abiotic facets because it is well-suited to analyzing large data sets and continuous data. We used the K-means clustering function within the R statistical software package [38], parameterized for 10,000 iterations, 20 random starts, and the “Hartigan-Wong” algorithm [39]. We used the Krzanowski-Lai Index to determine the optimal number of clusters. The Krzanowski-Lai index has been shown to perform well regardless of the underlying data structure, cluster number, and total number of input variables [40]. To gauge the sensitivity of our results to algorithm choice, we also defined clusters using an alternative clustering algorithm [41] and alternative numbers of clusters (Text S1, Figure S2).

We used a multivariate statistical approach to visualize the location and contemporary biotic associations of abiotic facets in the Columbia Plateau ecoregion. We constructed contingency tables to cross-tabulate the abiotic facets with contemporary vegetation cover from the National Vegetation Classification [42], [43]. We then performed a correspondence analysis on these contingency tables to evaluate the relationship between facets and contemporary vegetation. To minimize the influence of extreme outliers on our multivariate ordinations, we excluded some rare vegetation types from this analysis. We then produced ordination “joint plots,” in which close proximity in ordination space indicates more frequent co-occurrences of abiotic facets and vegetation types in the Columbia Plateau ecoregion.

### Biodiversity conservation elements

We used data describing plant associations and rare-species occurrences in accordance with a 1999 ecoregional conservation plan for the Columbia Plateau to create a reserve network based on current biodiversity elements [33]. The data, provided by The Nature Conservancy, consists of 93 coarse- and fine-filter elements, including 66 plant assemblages, such as Columbia Basin Palouse Prairie and Columbia Plateau Low Sagebrush Steppe, and 27 threatened or endangered plant and animal species. The species data includes bird, mammal, amphibian, mollusc, and vascular plant species listed as imperiled, threatened, endangered, or as aquatic target species. A few of the species include the Van Dyke's Salamander (*Plethodon vandykei*), Brewer's Sparrow (*Spizella breweri*), Ashy Pebblesnail (*Fluminicola fuscus*), Townsend's Big-eared Bat (*Corynorhinus townsendii*), and Northern Blue-eyed grass (*Sisyrinchium septentrionale*).

### Prioritizing areas for conservation

We prioritized planning units for a cost-efficient reserve network that met a set of goals for protecting certain quantities of the abiotic facets or biodiversity elements. We compared these two networks of priority planning units. Each prioritization was based on an optimization procedure and a measure of each planning unit's cost.

We used sixth-level (sub-watershed) hydrologic unit codes (HUC-12) [44] with an average size of 93.12 km^2^ (SD = 35.63 km^2^) for the 1200 planning units for prioritization (Figure 1). We calculated the area of each abiotic facet and the number of occurrences or areas of each biodiversity element within each HUC. Although the scale of these planning units is larger than the scale of land parcels available for purchase or easements, our goal was to identify priority planning units within the Columbia Plateau ecoregion within which to focus more local conservation efforts.

The current proportions and distributions of species may change in a changing climate, and we may not be able to anticipate the value of a certain facet type for future biodiversity conservation. Therefore, rather than targeting abiotic facets proportionally (as in traditional goal setting), we chose to target equal quantities of each abiotic facet. By doing so, we would create the maximally diverse template of all facet types. Therefore, we designated the goals for protection of each facet by dividing 15% of the total area of the ecoregion equally among all facet types. If the goal for a facet exceeded the total area of the facet, we required the protection of the entire facet. We aimed to represent 15% of the landscape to produce a network of reserves of roughly the same area as the network of reserves resulting from planning for biodiversity objectives. To explore the impact of these decisions on our results, we also prioritized planning units to represent a second set of goals based on the relative proportions of facets in the region according to the recommendation for goals for multi-scalar features [45] (Text S1, Figure S3).

We used biodiversity conservation goals established by The Nature Conservancy according to similar recommendations described in Tear et al. [46]. We required quantities of rare species based on their prevalence and 30% of the historical distribution of plant associations or all remaining occurrences if less than 30% of the historic distribution remained.

**Figure 2:**
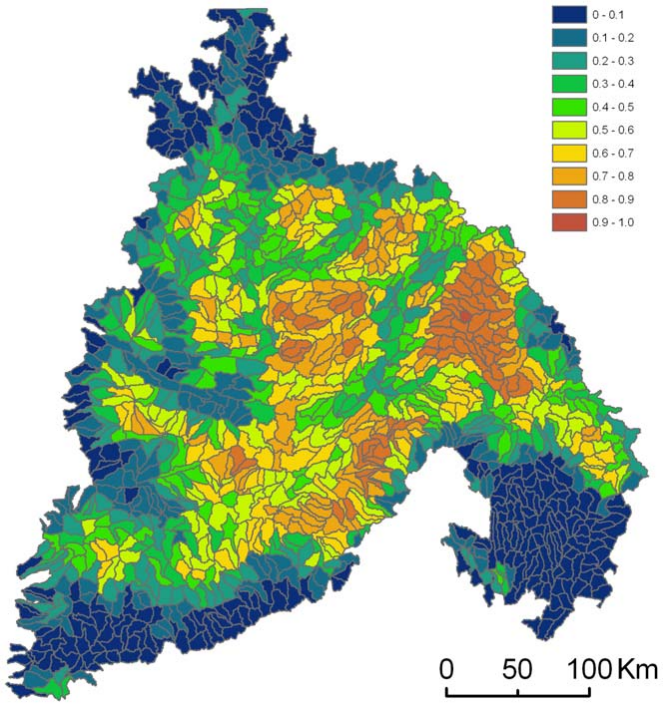
Planning unit cost based on naturalness. Planning units are ranked by the average degree to which each grid cell within the planning unit has been converted to human-dominated land-cover types (e.g. urban or agricultural areas). Planning units that, on average, have grid cells with less natural land cover remaining are red and have a high cost relative to areas with more natural land-cover types remaining (blue). The actual values are calculated as 1-(the mean of the naturalness values of each grid cell in the planning unit). Naturalness refers to the proportion of natural land-cover types in each grid cell.

Optimization procedures prioritize planning units that meet stated goals while minimizing the cost of the network. We assumed that the proportion of natural land-cover types (e.g., forests, sagebrush steppe, grasslands, etc.), hereafter referred to as naturalness [47], averaged across every grid cell in each planning unit is indicative of the ability of that planning unit to support natural communities, or inversely, with the cost of restoring the planning unit to natural conditions. We therefore calculated the cost for each planning unit as 1 minus the average value of naturalness of all grid cells in each planning unit (Figure 2). For grid cells without naturalness values (e.g., open water), we calculated the mean naturalness within a 10×10 cell window around the cell. To explore the impact of this cost metric on our results, we also prioritized sites with a uniform cost for all planning units (Text S1, Figure S3).

We used Marxan software [48], which employs a simulated annealing algorithm, to prioritize planning units for conservation that meet the most goals for the minimum cost [49]. The stochastic nature of the algorithm means that it can be used to produce several near-optimal solutions of a reserve-selection problem [49]. We ran Marxan 1,000 times for each set of facets or biodiversity elements to determine the frequency with which each planning unit was included in a network of priority planning units. This frequency of inclusion indicates the relative importance (irreplaceabity) of each planning unit to achieving conservation goals in an efficient network.

After prioritizing the networks of planning units to represent all facets and all biodiversity elements, we compared the results of the two analyses. We also quantified incidental representation [50] as the percentage of biodiversity-element goals that were achieved by the most efficient network of planning units (i.e., the solution that had the lowest total cost with the highest representation) chosen to meet abiotic goals. We tested whether observed incidental representation of biodiversity by the most efficient abiotic network was better than expected by chance using a random permutation test based on the same number of planning units (192).

### Planning for restoration

The longer temporal scale of planning for a changing climate provides the opportunity to include planning units that provide less value to current biodiversity due to current land uses, but that with restoration, may provide for biodiversity in the future. Therefore, we explored two other reserve-selection scenarios; one designed to preserve more natural sites and one that allowed for less natural planning units (i.e., potential sites for restoration). To develop these two scenarios, we defined two naturalness thresholds. These thresholds represented relatively unimpacted conditions (naturalness ≥80%) and conditions that might require restoration (naturalness ≥60%). We excluded facets in areas with naturalness values below these thresholds. We then prioritized planning units in each of the two datasets as above but without using a naturalness-based cost. The comparison of these networks highlights opportunities for restoration. For example, the planning units prioritized only in the analysis of unimpacted conditions represent the areas important for conservation if restoration is not an option. Conversely, units indentified only in the restoration scenario represent locations that a planner might consider if resources are available for restoration.

## Results

**Figure 3:**
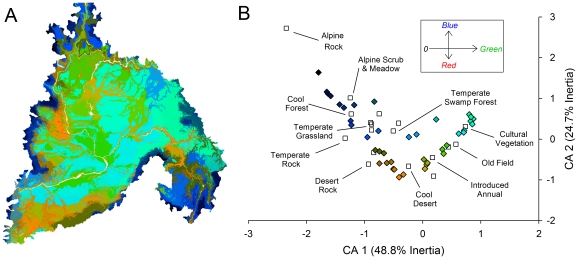
Abiotic facet distribution and correspondence with vegetation. This map depicts abiotic facets for the Columbia Plateau ecoregion (A). The ordination joint plot displays the correspondence between these abiotic facets (filled diamonds) and vegetation cover (open squares) (B). Select vegetation types are labeled in the ordination and abiotic facets are color-coded relative to their position on the ordination axes. Abiotic facets and vegetation types that occur in close proximity in the joint plot co-occur more frequently in the ecoregion.

**Figure 4:**
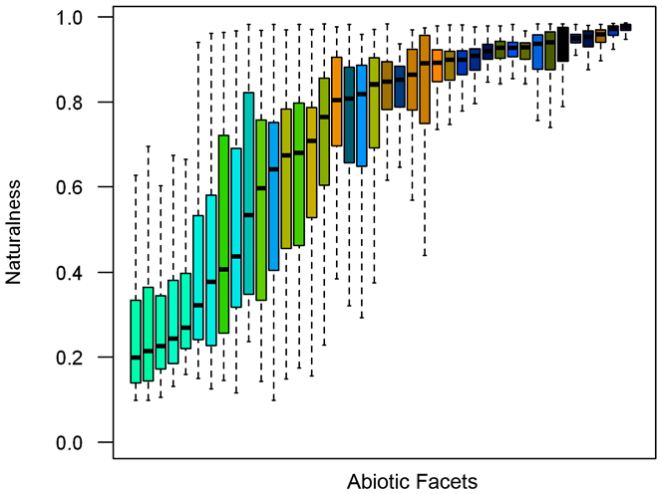
Boxplot of the naturalness of each facet. Each boxplot represents the naturalness (i.e., the proportion of natural land cover types) of each grid cell of each abiotic facet. The boxes are ordered on the x-axis by the increasing median naturalness of each facet (represented by the solid black line within each box) and are colored according to the relative positions of each facet on ordination axes from the correspondence analysis in Figure 3. Boxes represent the values between the 25th and 75th percentiles of naturalness of each abiotic facet. The dashed whiskers represent values that are 1.5 times the interquartile range or the most extreme value if no values exist beyond 1.5 times the interquartile range. Values that are more extreme than the dashed whiskers are considered outliers and are not graphed. The facet corresponding with open water is not graphed because naturalness values for open water were missing and were estimated based on neighboring grid cells for analysis.

We identified optimal aggregates of 41 clusters for abiotic facets based on the nine abiotic variables (Figure 3a). The correspondence analysis between the abiotic facets and vegetation cover were highly significant (χ^2^≥1669000, *p* <0.001), indicating that abiotic facets and vegetation were not independent of each other. Joint plots of the first two ordination axes (combined inertia of 73.5%) revealed clear ecological gradients in vegetation types and corresponding abiotic facets (Figure 3b). In general, the first correspondence analysis axis represented a gradient of soil depth and productivity, from shallow rocky soils to deeper productive soils largely converted to agricultural land uses. The second axis represented a gradient of elevation, temperature, and precipitation from higher, cooler, and wetter environments to low elevation arid lands. The facets also varied in their levels of naturalness (Figure 4). In general, facets at low elevations with deep, productive soils existed in less natural areas. The percentage of area of each facet that was targeted for protection (i.e., the goal quantities) also varied by facet with the cooler, moister alpine areas having goals that approached the total areas of the facets.

**Figure 5:**
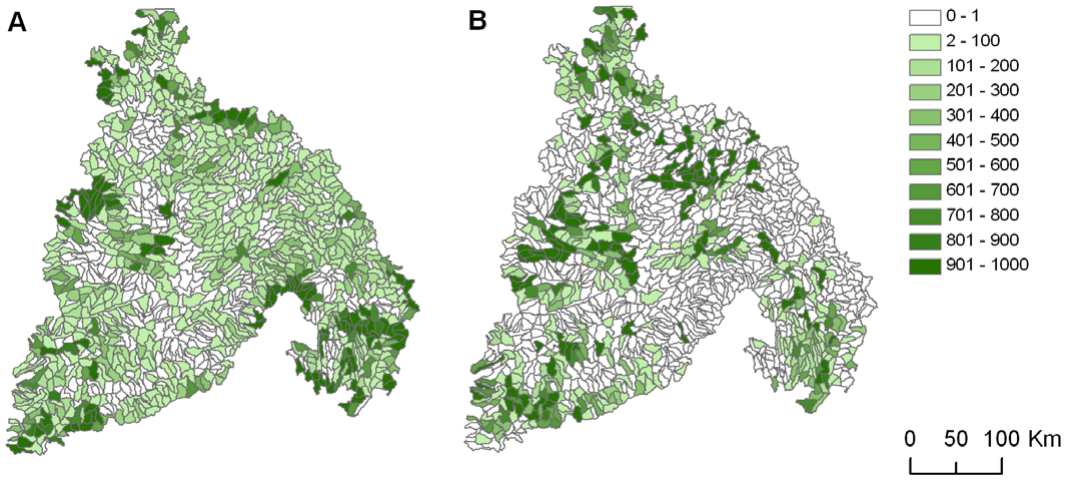
Irreplaceability of planning units. Irreplaceability is measured as the number of times a planning unit was selected across 1000 potential networks. Irreplaceablilty values are mapped for abiotic elements (A) and biodiversity elements (B).

**Figure 6:**
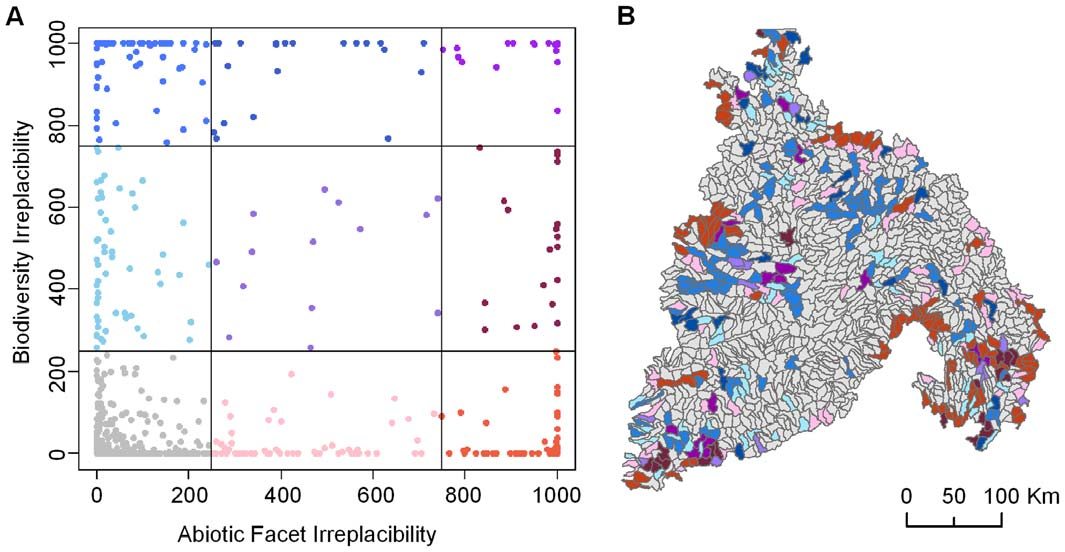
Scatterplot of irreplaceability. Irreplaceability is measured as the number of times a planning unit was selected across 1000 potential networks. The plot shows the irreplaceability of planning units for representing bioidiversity elements plotted against the irreplaceability of planning units for representing abiotic facets. The solid lines indicate divisions for low irreplaceability (i.e., selected in less than 250 solutions) or high irreplaceability (i.e, selected in more than 750 solutions) (A). The map displays the spatial location and irreplaceability values of planning units in the Columbia Plateau ecoregion corresponding to irreplaceability values for both target types (B). Blue planning units have high irreplaceability for biodiversity elements, red planning units have high irreplaceability for abiotic facets, and intermediate purple planning units have high irreapleacbility for both. Grey planning units are not important in representing either type of conservation element.

Prioritizing planning units based on facets and alternatively on biodiversity elements resulted in markedly different rankings of the units (Figure 5). The prioritization based on abiotic facets resulted in many more planning units of intermediate irreplaceability (i.e., units that were included in several, but not all networks), whereas the biodiversity-based prioritization ranked some sites as highly irreplaceable, many as not very irreplaceable, and few as intermediately irreplaceable. In addition, abiotic facet units with high irreplaceability were often located at the margins of the Columbia Plateau ecoregion, where greater topographic or climate variability associated with foothills of adjacent mountains may have driven prioritization in our reserve selection process. Such transitional planning units between ecoregions may be extremely valuable in conservation planning for climate change, as they may allow species to follow preferred climates to higher elevations over time. By contrast, biodiversity units with high irreplaceability were more often located in the interior of the Columbia Plateau ecoregion, likely in response to the current habitats and ranges of targeted rare species and vegetation types. Despite these differences, some planning units were irreplaceable for both abiotic facets and biotic elements, and many planning units were of minimal importance in achieving either objective (Figure 6).

The most efficient network of planning units selected to represent abiotic facets also incidentally represented 59% of biodiversity elements. This representation is significantly better than that expected from random permutations of the same number of planning units (mean of 47% of elements represented in 1000 permutations, p = 0.0345). However, some types of biodiversity elements were better represented than others. The most efficient network of units selected to represent the abiotic facets represented 76% of the plant assemblages (coarse filter), but only 16% of the rare species (fine filter).

**Figure 7:**
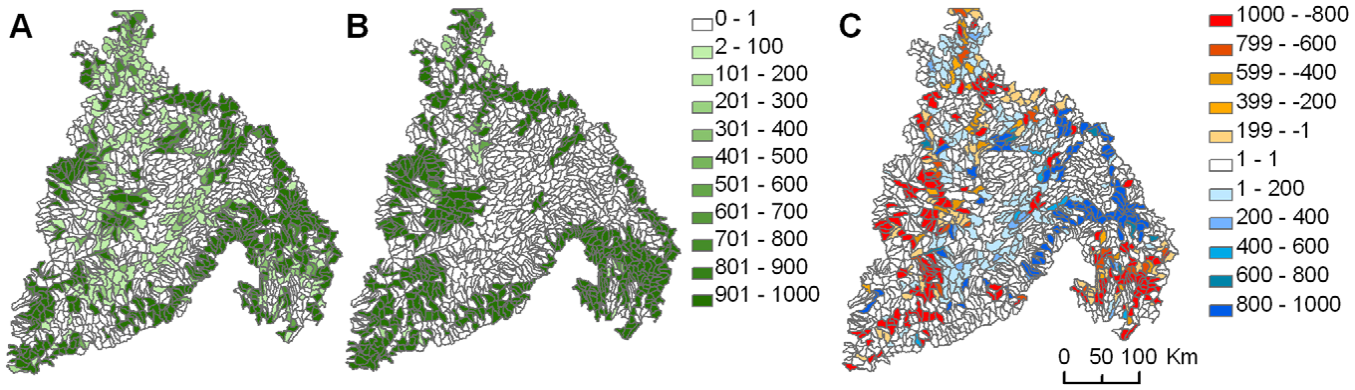
Comparison of irreplaceability of planning units in the restore vs. protect scenarios. Irreplaceability is measured as the number of times a planning unit was selected across 1000 potential networks. Irreplaceablilty values are mapped for networks prioritized to represent abiotic elements restricted to ≥60% naturalness (i.e., grid cells with ≥60% natural land types) (A) and ≥80% naturalness (B). The differences in irreplaceability values between the two networks (A and B) are mapped (C). Blue areas indicate additional conservation opportunities in the network when restoration is an option for the region.

Many additional planning units could contribute to a network of sites that would protect the abiotic diversity of the Plateau if major restoration efforts are undertaken. Some of the more impacted sites that could significantly contribute to the goal of protecting abiotic diversity are in the eastern part of the Plateau (Figure 7). The two prioritization scenarios that we used to identify these potential restoration sites differed in the area of land that was available for inclusion in a network of priority sites. Excluding areas with naturalness values below 80% (protection scenario) left just 40% of the landscape available for protection whereas excluding areas below 60% naturalness (restoration scenario) left 59% of the landscape available for protection or restoration. Not surprisingly, planning units ranked by the protection scenario were more highly irreplaceable (i.e., had fewer alternatives for achieving goals) whereas networks based on the restoration scenario had more flexibility (i.e., more alternatives for achieving goals) (Figure 7 A and B).

## Discussion

Conservation plans that focus solely on the distribution of today's biota are prone to fail in a changing climate. As species move, communities will be reshuffled and ecosystems will be altered—the areas that maximally protect today's biodiversity may fail to protect tomorrow's biodiversity. Conserving the abiotic stage on which future biodiversity plays out will be an important part of a conservation-planning strategy for addressing climate change [23], [24], [51], [52].

There will be challenges to shifting the focus of conservation planning to include abiotic landscape elements. Conservation planners have traditionally prioritized areas based on biotic elements, for which one can more easily garner public support. Furthermore, many environmental laws, such as the U. S. Endangered Species Act, apply to species, populations, and the habitats that currently sustain them rather than to physical features. Therefore, allocating resources to the protection of abiotic facets may be less appealing to conservation funders or policy makers. Nonetheless, planning for both biotic and abiotc diversity is not a novel concept [29], [51], [53], and the arguments for doing so, particularly in light of climate change, are clear.

The allocation of conservation resources to abiotic facets may be less objectionable if facets are integrated into the coarse- and fine-filter planning framework as a surrogate for coarse-filter elements [24]. The concept of conserving fine-filter and coarse-filter elements of biodiversity has already gained widespread acceptance as an important methodology in conservation planning [3], [54], [55]. Fine-filter elements are typically rare, threatened, or important focal species. Coarse-filter elements are commonly vegetation-based ecological communities or systems that serve as proxies for species for which detailed species-distribution information is lacking. As one might expect, planning units selected to represent abiotic facets in the Columbia Plateau poorly represented fine-filter biotic elements but adequately represented coarse-filter biotic elements. Although the lack of representation of the rare species is in part explained by the fact that many rare species will be left out by a coarse-filter approach, this lack of representation may also reflect deficiencies in the individual species distribution data or the fact that much of the habitat and historical distributions of these sepecies has been lost. The general deficiencies in species distribution data are well known and well documented [56], [57]. In the Columbia Plateau, data were were opportunistically collected. Therefore, sites that are more difficult to access, such as private lands and more remote areas, are likely to be undersampled.

### Using abiotic-facet-based planning to inform restoration strategies

Traditionally, restoration efforts have focused on returning a system to some former state. That simple goal, like the goal of protecting the current distribution of biodiversity, is challenged by climate change [58], [59], [60]. In the face of climate change, restoration will need to look forward, not back [58], [61]. Although less impacted areas are typically preferred for present-day planning, planning units that require active restoration may be suitable for a facet-based reserve network over a long time frame even if their current ecological integrity or their ability to support biota is not presently ideal. We found that by including planning units in which ecosystems were more heavily altered by human activities dramatically increased the flexibility (the ability to substitute one planning unit for another to fulfill conservation goals) of the network of planning units in the Columbia Plateau ecoregion (Figure 7). This flexibility is important for achieving conservation objectives in such a human-dominated landscape. Because of the heavy agricultural development of the Columbia Plateau ecoregion, putting resources towards restoration may be the only way to protect that component of the ecological stage.

### Implications for conservation planning

There are several ways in which abiotic elements could be integrated into a conservation-planning process. Perhaps most simply, one could take a prioritization based on abiotic facets and use it to refine an existing prioritization based on more traditional biotic elements. For example, when deciding between two potential land purchases of areas that provide relatively similar biodiversity benefits, one might select the area that contributes more to the goal of representing the diversity of abiotic elements. Using such an approach, the planning units in the Columbia Plateau with high abiotic and high biotic irreplaceability (the purple symbols in Figure 6) would be given a higher priority than the units that were only highly irreplaceably with respect to biotic elements (the blue symbols in Figure 6).

Including abiotic facets in the conservation-planning process will very likely increase the robustness of a protected area network to climate change. However, protecting abiotic facets alone will be unlikely to adequately address the impacts of climate change on biodiversity. First, as noted above, abiotic facets can serve as a coarse filter in the conservation-planning process, however, rare species with more specific habitat requirements or ecological needs may not be represented by coarse-filter networks. Thus, some effort will be required to address the needs of fine-filter elements of biodiversity in a changing climate.

The few examples of conservation planning for fine-filter elements in a changing climate make use of forecasts of shifts in species' distributions. Bioclimatic models that use the relationships between species distributions and current climate in conjunction with future climate projections are common tools for anticipating shifts in species ranges [62]. However, there are many uncertainties associated with these projections and the broad scale of bioclimatic model projections may not be implementable at the scale of ecoregional planning [63]. Process-based models that use species' physiological responses to climate variables or spatially explicit population models (SEPMs) linked with dynamic models of vegetation, hydrology, or fire may be used to forecast species responses to climate change at a more appropriate scale for conservation planning [64]. However, the data required to parameterize these models is lacking for all but a few species. Conservation planning and allocation of resources based on these uncertain forecasts may be risky, but these methods may be useful tools for conserving rare species and specialists in a changing climate [65].

A second reason that protecting abiotic facets may be a necessary but insufficient approach to addressing climate change in the conservation-planning process is that it does not account for the fact that species will need to move across the landscape to reach these physical environments. If these areas are to act as an ecological stage on which new communities and new ecosystems will assemble, then species will need to be able to move among them. Thus, providing connectivity will be a critical part of the conservation-planning process for addressing climate change. Not surprisingly, increasing landscape connectivity is one of the most-often cited adaptation strategies for protecting biodiversity in a changing climate [16]. Although planning for landscape connectivity has traditionally focused on linking currently occupied habitats for certain species [27], more recent connectivity planning has focused on connecting abiotic elements that facilitate climate-change induced movement for a variety of species. In association with the land facet approach, linkages can be identified along contiguous facet types or in areas of high local facet diversity [66]. Software tools have been developed to design such linkages [24], [67].

Incorporating facet-based planning into existing conservation-planning frameworks has the potential to make reserve networks more robust to climate change. Nonetheless, protecting abiotic facets alone will likely prove to be an ineffective tactic for protecting biodiversity in a changing climate. A more effective approach will likely include a balance of protecting abiotic and biotic elements, planning for forecasted climate impacts on particularly vulnerable species, and increasing the connectivity of the landscape.

## Supporting Information

Text S1 Sensitivity of the abiotic-facet approach to procedural decisions. The methods for facet designation and prioritization require one to make relatively subjective decisions in defining the facets and prioritizing the planning units. This supporting document describes the sensitivity of the results to alternative decisions in the analytical process such as using different combinations of input variables, a different clustering algorithm (Lloyd), a different goal-setting process (proportional), or a different cost for planning units (uniform).
(DOC)

Figure S1 **Climate facet and land facet distribution and correspondence with vegetation.** These maps depict the facets clustered from the four climate variables only (A) or the five land variables only (C) projected to the Columbia Plateau ecoregion. The ordination joint plots display the correspondence between the climate facets (B) or land **f**acets (D) (filled diamonds) with vegetation cover (open squares). Select vegetation types are labeled in ordinations and abiotic facets (filled diamonds) are color-coded relative to their positions on the ordination axes. Abiotic facets and vegetation types that occur in close proximity in the joint plot co-occur more frequently in the ecoregion.
(TIF)

Figure S2 **Abiotic facet distribution of Lloyd's clustering algorithm and correspondence with vegetation.** This map depicts abiotic facets designated by Lloyd's clustering algorithm projected to the Columbia Plateau ecoregion (A). The ordination joint plot displays the correspondence between (B) these abiotic facets (filled diamonds) and vegetation cover (open squares). Select vegetation types are labeled in the ordination and abiotic facets are color-coded relative to their position on the ordination axes.
(TIF)

Figure S3 **Comparison of irreplaceability of planning units resulting from various analytical process decisions.** Irreplaceability is measured as the number of times a planning unit was selected across 1000 potential networks. Irreplaceability values are mapped for networks that are based on different input parameters that were determined by decisions made during the analytical process. The baseline map of irreplacebility represents the network resulting from the Hartigan-Wong algorithm for facet designation, objectives based on equal-area goals, and planning unit cost based on naturalness (i.e., the proportion of natural landcover averaged across each grid cell in a planning unit). Each subsequent column represents a single alternative decision incorporated with the other baseline decisions (i.e. a different clustering algorithm (Lloyd), goal-setting process (proportional), or cost of planning units (uniform)). The rows represent the irreplaceability values resulting from these decisions and different combinations of input variables.
(TIF)

Table S1 Data Sources. The sources and information for the data layers used in the analysis.
(DOC)
