# Supplementary material for: Systematic Conservation Planning in the Face of Climate Change: Bet-Hedging on the Columbia Plateau
Source: PLoS One. 2011 Dec 8;6(12):e28788. doi: 10.1371/journal.pone.0028788 (PMC3234274; doi:10.1371/journal.pone.0028788)
Supplement: Text S1 — Sensitivity of the abiotic-facet approach to procedural decisions. The methods for facet designation and prioritization require one to make relatively subjective decisions in defining the facets and prioritizing the planning units. This supporting document describes the sensitivity of the results to alternative decisions in the analytical process such as using different combinations of input variables, a different clustering algorithm (Lloyd), a different goal-setting process (proportional), or a different cost for planning units (uniform). (DOC) [file pone.0028788.s001.doc]

**Sensitivity of the abiotic-facet approach to procedural decisions**

There are many aspects of the approach presented here that require one to make relatively subjective decisions in defining the facets and prioritizing the planning units. The facets as well as the specific ranking of planning units are sensitive, to varying degrees, to these particular choices made during the analytical process. First, we aggregated data to 240m grid cells in order to decrease processing time in the cluster analysis. Although some local variation is lost in the averaging process, the majority of this variation occured in zones of transition on the borders of the ecoregion and therefore likely did not have a large influence on overall facet designation in the Columbia Plateau. For example, across the ecoregion, 98% of 30m elevation values were within 15 meters of the value represented by the aggregated 240m grid cell.

Alternatively, the choice of which varaibles to include in the definition of facets can have a significant effect on the facets produced. Clusters based on all of the abiotic variables resulted in 41 optimal clusters. Clusters based on only the 5 topographic and soil variables resulted in 48 optimal clusters that had a similar distribution to the 41 clusters, and clusters based on only the four climate variables resulted in 44 optimal clusters that resutled in a more aggregated distribution with less regional variability. The reduced variability in the facets based on climate variables alone may not be distinguishing enough to be useful for planning unit prioritization in the Columbia Plateau. Despite the differences in the number and distribution of facets, the correspondence of all sets of clusters with vegetation were still highly significant (Figure S1).

The selection of clustering algorithms also affected the resulting facets. Using Lloyd’s clustering algorithm to cluster the 9 abiotic variables resulted in 51 abiotic facets, in contrast to the 41 facets designated with the Hartigan-Wong algorithm. Despite the difference in the number of facets, the sets of facets produced by the two clustering algorithms were qualitatively similar (Figure S2). Other clustering algorithms and indices for identifying optimal cluster numbers exist and may result in different numbers of clusters or distributions of facets.

The differences in the facets produced by different input variables or clustering algorithms resulted in some large differences in the priority assigned to specific planning units but had very little impact on the overall pattern of the resulting planning unit prioritization (Figure S3). Conversely, goal setting is highly subjective and had a large impact on the prioritization of planning units (Figure S3). For example, the equal-area goal-setting approach we used was vulnerable to the inadvertent prioritization of the alpine facets at ecoregion margins that are not characteristic of the ecoregion. These rare facets may be sufficiently represented and even common in a bordering ecoregion. Therefore, goals should be determined at a broader scale that spans ecoregional boundaries and adjusted accordingly within the ecoregion. Alternatively, setting goals as weighted percentages avoided this disproportionate prioritization at the ecoregion boundaries (Figure S3).

Assigning costs to each planning unit based on naturalness also had a large impact on planning-unit prioritization. Ignoring cost altogether reduced the irreplaceability of many planning units (Figure S3) emphasizing that highly irreplaceable planning units in the network prioritized with naturalness as a cost likely contain the few remaining natural remnants of particular facets**.**

Lloyd, S. P. 1982. Least-Squares Quantization in PCM. IEEE Transactions on Information Theory **28**:129-137.
