# Supplementary figures and images for: Systematic Conservation Planning in the Face of Climate Change: Bet-Hedging on the Columbia Plateau
Source: PLoS One. 2011 Dec 8;6(12):e28788. doi: 10.1371/journal.pone.0028788 (PMC3234274; doi:10.1371/journal.pone.0028788)

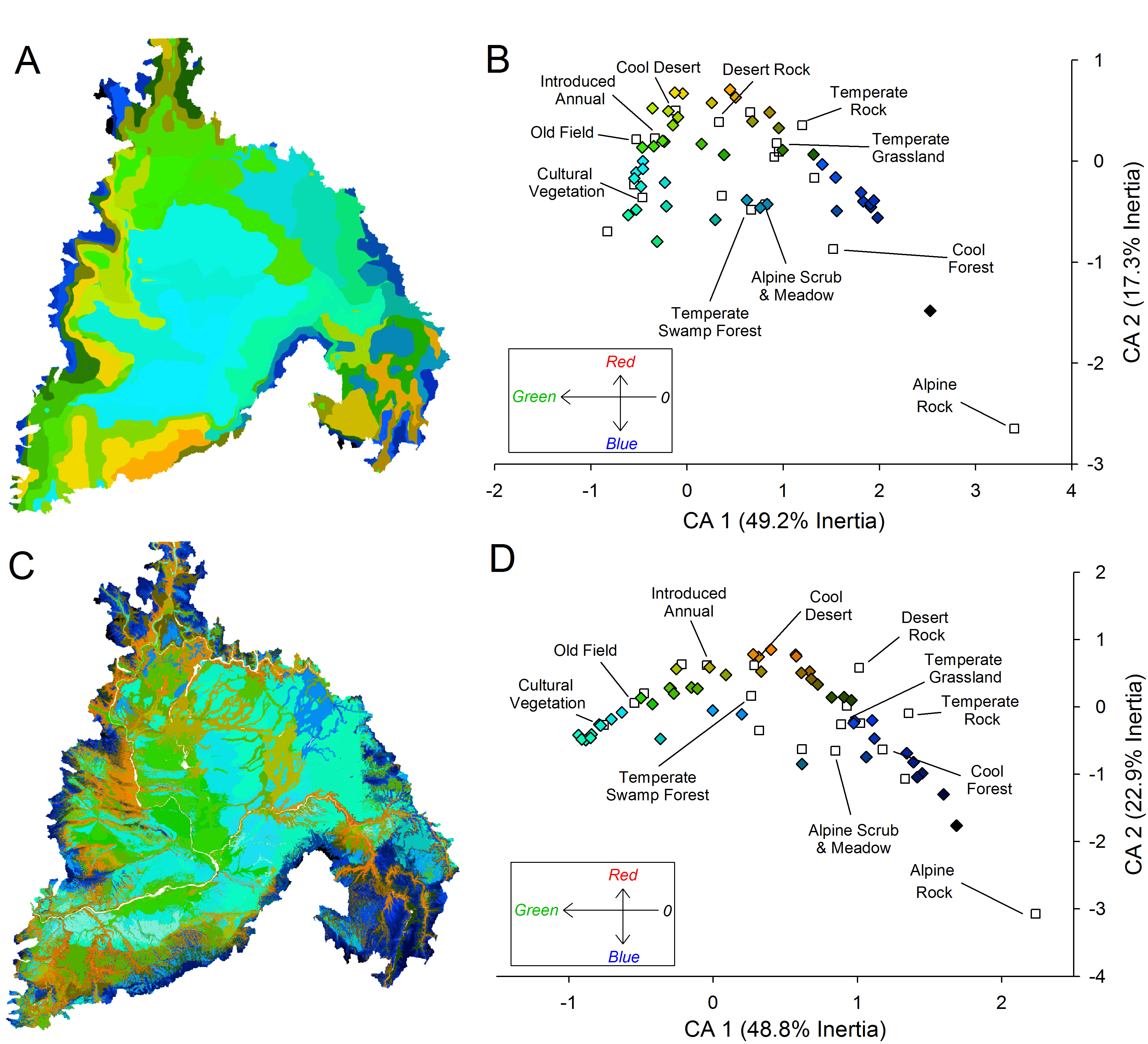

Supplement: Figure S1 — Climate facet and land facet distribution and correspondence with vegetation. These maps depict the facets clustered from the four climate variables only (A) or the five land variables only (C) projected to the Columbia Plateau ecoregion. The ordination joint plots display the correspondence between the climate facets (B) or land facets (D) (filled diamonds) with vegetation cover (open squares). Select vegetation types are labeled in ordinations and abiotic facets (filled diamonds) are color-coded relative to their positions on the ordination axes. Abiotic facets and vegetation types that occur in close proximity in the joint plot co-occur more frequently in the ecoregion. (TIF) [file pone.0028788.s002.tif]

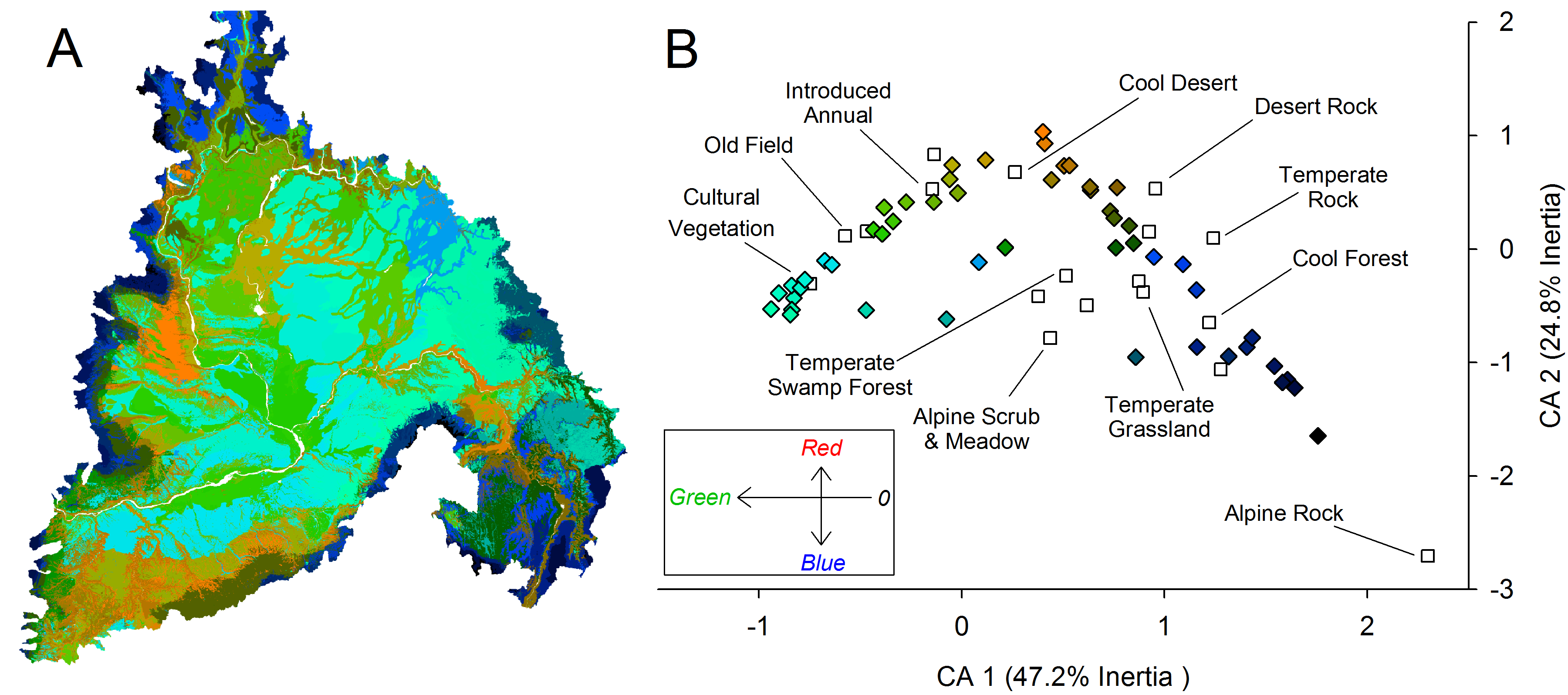

Supplement: Figure S2 — Abiotic facet distribution of Lloyd's clustering algorithm and correspondence with vegetation. This map depicts abiotic facets designated by Lloyd's clustering algorithm projected to the Columbia Plateau ecoregion (A). The ordination joint plot displays the correspondence between (B) these abiotic facets (filled diamonds) and vegetation cover (open squares). Select vegetation types are labeled in the ordination and abiotic facets are color-coded relative to their position on the ordination axes. (TIF) [file pone.0028788.s003.tif]

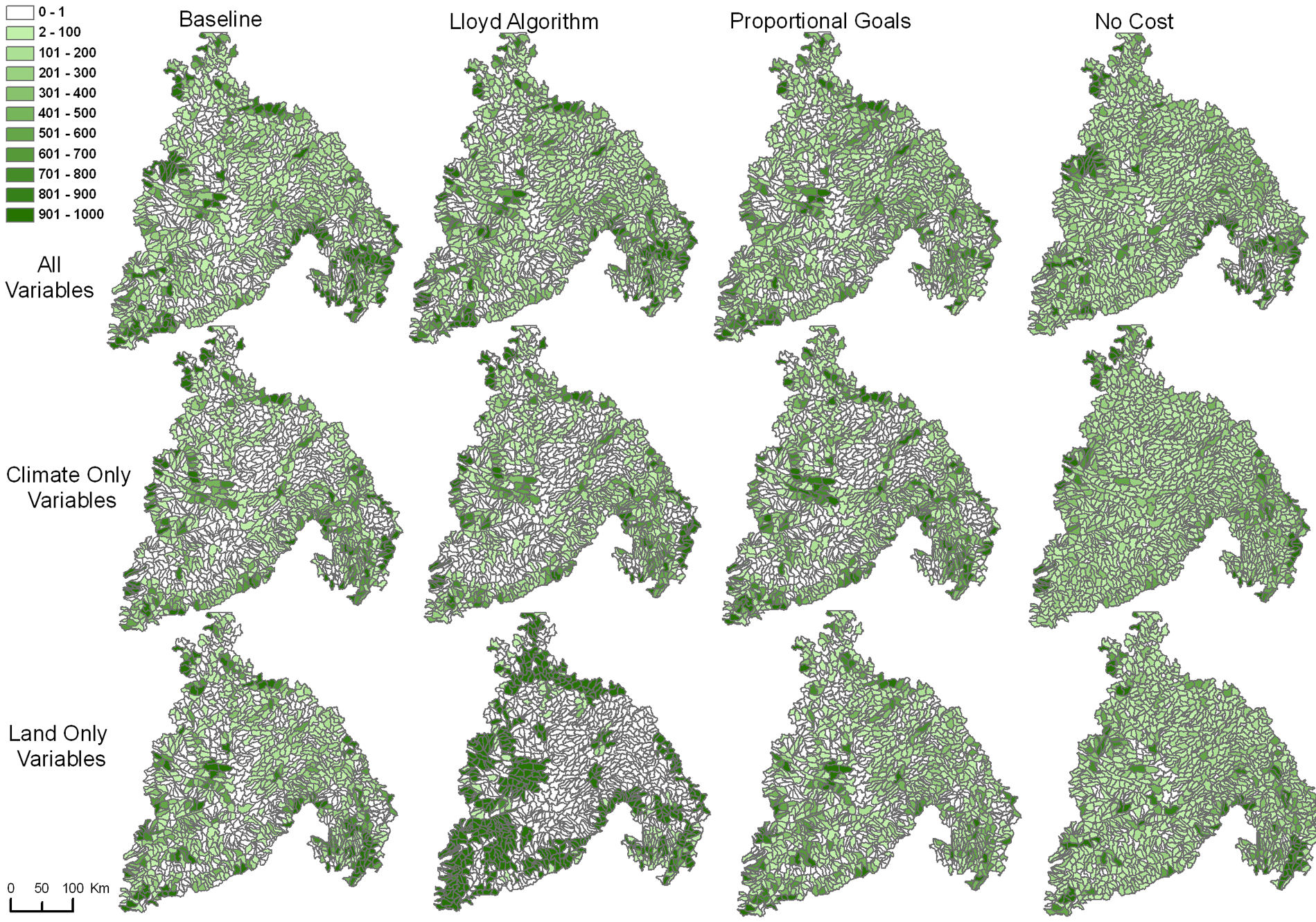

Supplement: Figure S3 — Comparison of irreplaceability of planning units resulting from various analytical process decisions. Irreplaceability is measured as the number of times a planning unit was selected across 1000 potential networks. Irreplaceability values are mapped for networks that are based on different input parameters that were determined by decisions made during the analytical process. The baseline map of irreplacebility represents the network resulting from the Hartigan-Wong algorithm for facet designation, objectives based on equal-area goals, and planning unit cost based on naturalness (i.e., the proportion of natural landcover averaged across each grid cell in a planning unit). Each subsequent column represents a single alternative decision incorporated with the other baseline decisions (i.e. a different clustering algorithm (Lloyd), goal-setting process (proportional), or cost of planning units (uniform)). The rows represent the irreplaceability values resulting from these decisions and different combinations of input variables. (TIF) [file pone.0028788.s004.tif]
