# Supplementary material for: Systematic Conservation Planning in the Face of Climate Change: Bet-Hedging on the Columbia Plateau
Source: PLoS One. 2011 Dec 8;6(12):e28788. doi: 10.1371/journal.pone.0028788 (PMC3234274; doi:10.1371/journal.pone.0028788)
Supplement: Table S1 — Data Sources. The sources and information for the data layers used in the analysis. (DOC) [file pone.0028788.s005.doc]

Table S1. **Data Sources.** The sources and information for the data layers used in the analysis.

| **Data** | **Source** | **Information** |
| --- | --- | --- |
| DEM – Idaho | Idaho Geospatial Data Clearinghouse 2004 | Data originally derived from USGS 7.5 minute elevation data |
| DEM – Oregon | USGS 1999 [2] | Data originally derived from USGS 7.5 minute elevation data |
| DEM - Washington | Geomorphological Research Group 2001 [3]; Washington Department of Natural Resources 2002 [4] | Data originally derived from USGS 7.5 minute elevation data |
| Soil Data - county | Soil Survey Geographic (SSURGO); Soil Survey Staff 2010 [5] | Data were available at scales of 1:12,000 or 1:63,360. We gridded the data using maximum combined area for cell assignment. |
| Soil Data - country | State Soil Geographic (STATSGO); Soil Survey Staff 2010 [6] | Data were available at scale of 1:250,000 . We gridded the data using maximum combined area for cell assignment. |
| Climate Data | Climate Impacts Group at the University of Washington in collaboration with the WA State Department of Ecology, Bonneville Power Administration, Northwest Power and Conservation Council, Oregon Water Resources Department, and the B.C. Ministry of the Environment [7] | Data were downloaded from the Columbia Basin Climate Change Scenarios Project website. Climate data originally came from the National Climatic Data Center (NCDC), Cooperative Observers (COOP) network, and Environment Canada (EC) and are summarized at monthly time steps at 1/16th degree latitude by longitude resolution across the study area (approximately 30 km^2^ per cell) and averaged for the time period 1915-2006 |
| Biodiversity Data | The Nature Conservancy | Occurrence data for freshwater and terrestrial species and plant associations. Quantities are reported in: acres for vegetation, number of occurrences for terrestrial species, and linear meters for aquatic species distributions |

1. Idaho Geospatial Data Clearinghouse (2004) Digital elevation of Idaho with a horizontal grid spacing of 30-meters: Idaho Geospatial Data Clearinghouse, Moscow, ID USA. Available: <http://cloud.insideidaho.org/webMaps/flash/tiledownload/index.html?collection=elevation&layerName=1999_30m_Idaho>. Accessed 2010 Feb 9.

2. USGS, Oregon Geospatial Data Clearinghouse and the Oregon Department of Environmental Quality (DEQ) (1999) Oregon 10m DEM. Available: <http://buccaneer.geo.orst.edu/dem>. Accessed 2010 Feb 9.

3. Geomorphological Research Group (2001) Mosaicked 30-meter Data for Eastern (Zone 11) Washington. Available: <http://gis.ess.washington.edu/data/raster/thirtymeter/index.html>. Accessed 2010 Feb 9.

4. Washington Department of Natural Resources (2002) DEM30. Available: [http://www3.wadnr.gov/dnrapp10/data/dataweb/dmmatrix.html](http://fortress.wa.gov/dnr/app1/dataweb/metadata/Public Access at: http:/www3.wadnr.gov/dnrapp10/data/dataweb/dmmatrix.html). Accessed 2010 Feb 9.

## 5. Soil Survey Staff, Natural Resources Conservation Service, United States Department of Agriculture. Soil Survey Geographic (**SSURGO**) Database for Washington, Idaho, Oregon. Available: [**http://soildatamart.nrcs.usda.gov**](http://soildatamart.nrcs.usda.gov/). Accessed 2010 Feb 9.

## 6. Soil Survey Staff, Natural Resources Conservation Service, United States Department of Agriculture. U.S. General Soil Map (**STATSGO2**) for Washington, Idaho, Oregon. Available: [**http://soildatamart.nrcs.usda.gov**](http://soildatamart.nrcs.usda.gov/). Accessed 2010 Feb 9.

7.Climate Impacts Group (2011) Columbia Basin Climate Change Scenarios Project (PI: Alan F. Hamlet), University of Washington, Available: <http://www.hydro.washington.edu/2860/>. Accessed 2011 May 24.
